# INTERVENTIONS IN SUBACUTE PAEDIATRIC INPATIENT NEUROREHABILITATION: AN UMBRELLA REVIEW

**DOI:** 10.2340/jrm.v57.42328

**Published:** 2025-04-02

**Authors:** Ivan ROBERTSON, Denise TAYLOR, Jimmy CHONG

**Affiliations:** 1 Department of Women’s and Children’s Health, Waikato Hospital, Te Whatu Ora Waikato, Hamilton; 2 Health & Rehabilitation Research Institute, Auckland University of Technology, Auckland; 3 Starship Paediatric Rehabilitation Service, Te Whatu Ora Te Toka Tumai Auckland, Auckland, New Zealand

**Keywords:** neurological rehabilitation, paediatric, hospital, rehabilitation, subacute care

## Abstract

**Background:**

Rehabilitation is vital for optimizing recovery following neurological injuries. However, much of what is done for the paediatric population relies heavily on the adult literature or is based on expert opinion. No previous attempt has been made to collate high-quality evidence pertaining to the subacute period.

**Objectives:**

To summarize and appraise the evidence from systematic reviews regarding the efficacy of inpatient neurorehabilitation interventions for paediatric patients in the 6 months immediately following neurological injury.

**Method:**

A systematic search was conducted of PubMed, Ovid Medline, Cumulative Index to Nursing and Allied Health Literature, Embase, and Cochrane databases, as well as using Google Scholar. Selection required an appraisal of efficacy of an inpatient rehabilitation intervention delivered within 6 months of injury.

**Results:**

1,250 papers were found. Titles and abstracts were compared against the selection criteria, with 52 papers being selected for full-text review. None of these met the selection criteria. Exclusions were often due to setting and chronicity.

**Conclusions:**

This review reveals a critical lack of systematically reviewed evidence within the scope investigated. There is a pressing need for evaluation of current interventions. In the future, alternative approaches to identifying and appraising different forms of available evidence could be considered.

## LAY ABSTRACT

This review aimed to assess the effectiveness of hospital-based rehabilitation treatments for children recovering from neurological injuries in the early months following their injury. Although rehabilitation is crucial for recovery, most practices for children are based on expert opinion. The researchers searched multiple databases to find reviews that systematically outlined and evaluated studies in this space. Of 1,250 studies, none met the criteria for inclusion in this review. This highlights the need for more research specifically focused on children, to ensure they receive the most effective care.

Paediatric neurorehabilitation is a critical component of care for children recovering from neurological injuries, such as traumatic brain injuries, strokes, and spinal cord injuries. These conditions often result in significant physical and cognitive impairments that adversely impact development and quality of life. Early, effective neurorehabilitation is considered essential for optimizing recovery and reducing the risk and severity of long-term disabilities (1).

According to the World Health Organization, rehabilitation is defined as “a set of interventions designed to optimise functioning and reduce disability in individuals with health conditions in interaction with their environment” (2). For the purposes of this review, we define “neurorehabilitation” as rehabilitation primarily directed towards optimizing deficits from insults to the neurological system. The “sub-acute” phase is the period following primary treatment when medical stability is well established. In severe and some moderate cases, this is typically but not exclusively when patients are suitable for transfer to a dedicated rehabilitation unit.

Rehabilitation within the subacute phase requires careful consideration due to the physiological changes that occur during this period, which can significantly influence recovery outcomes. Effective rehabilitation during this phase can enhance neuroplasticity and functional recovery, making it a crucial period for intervention (3).

Much of what is done for the paediatric population in this domain relies heavily on expert opinion (4) and extrapolation from practice for adults. Children’s neurodevelopmental processes and responses to rehabilitation likely differ significantly from those of adults. Whereas adult rehabilitation is often aimed at a return to baseline, for children the goal is to minimize the impact on their developmental trajectory. Despite neuroplasticity, early disruption to development may have greater long-term effects than later disruption (5, 6). Additionally, the modality of intervention delivery needs to be developmentally appropriate. Because of differences in neurophysiology, the pathophysiology of injury in children is different from comparable injuries in adults (7). As such, it is important to base guidelines on evidence relevant to the paediatric population.

No previous attempt has been made to collate high-quality evidence pertaining specifically to the sub-acute period in paediatric neurorehabilitation. A comprehensive evaluation of neurorehabilitation interventions is crucial. It is necessary for clinicians to understand which approaches are most effective for different types of neurological injuries and patient needs. Additionally, the relative effectiveness of interventions needs to be understood. There is a concerning lack of high-grade evidence underpinning current guidelines (4), and the hope is that consolidating what is available would inform treatment approaches and provide clarity around gaps future research needs to address.

Viewing rehabilitation through the lens of the International Classification of Functioning, Disability and Health: Children & Youth Version (ICF-Y) framework provides a valuable perspective, as it emphasizes not just the treatment of diseases but the optimization of overall functioning and participation in society. The ICF-Y framework allows for a comprehensive and structured approach to rehabilitation, ensuring that all aspects of a child’s functioning – physical, cognitive, and social – are considered. The use of ICF-Y can serve as a roadmap for structuring rehabilitation goals and interventions, facilitating improved communication among multidisciplinary teams, and ensuring that rehabilitation efforts are aligned with the child’s long-term developmental needs (8, 9).

This review seeks to address the need for evidence-based interventions in 1 arena of paediatric rehabilitation. An umbrella review, also known as a systematic review of systematic reviews, aims to consolidate evidence from existing systematic reviews and meta-analyses on a particular topic (10). By providing a broader synthesis of findings, umbrella reviews are useful in fields like paediatric neurorehabilitation, where the evidence base is complex and multifaceted, encompassing various interventions and outcomes. We shall focus exclusively on the state of evidence from systematic reviews for paediatric neurorehabilitation interventions in the inpatient setting during the subacute period. This umbrella review represents a novel and timely effort to consolidate the available evidence, providing much-needed clarity regarding the evidence-base for the future.

The primary objective of this review was to summarize and appraise the evidence from systematic reviews regarding the efficacy of inpatient neurorehabilitation for paediatric patients in the subacute phase after neurological injury or insult. This review will critically evaluate the quality, scope, and conclusions of the existing evidence, with the goal of offering insights into both well-established interventions and areas where further research is necessary.

## METHODOLOGY

This umbrella review was performed in accordance with the Protocol submitted to Prospero (11) under the same title and authorship.

### Search strategy

A comprehensive search of peer-reviewed published literature was conducted using healthcare-related databases, namely PubMed, Ovid Medline, Cumulative Index to Nursing and Allied Health Literature, Embase, Cochrane Database of Systematic Reviews, and Google Scholar.

There is a large range of potentially relevant neurological terms, and it is possible that relevant papers may not use broad terms in their titles or abstracts. To avoid overly complex searches and to avoid missing reviews investigating rare conditions, rehabilitation generally was included in the search strategy without limiting it using neurological terms. Similarly, parameters to filter for the subacute phase of injury were not included, given that some papers may not include specific reference to this in titles or abstracts.

It is acknowledged that, within paediatrics, a broad range of stages of development and rehabilitative needs are represented. While findings that diverge from evidence for adults would be expected to be most common in younger age groups, an age range was adopted that aligns with the paediatric literature at large, and that approximately corresponds to the ages seen by inpatient paediatric rehabilitation centres in the researchers’ locality.

Search strategies for each database were developed with assistance from Middlemore Hospital Library staff. These are outlined in Table I.

**Table I:** Search strategy: double columns under database headings indicate where searches were run twice with different approaches

Database	PubMed	Cochrane		Ovid Medline		CINAHL		Embase	Google Scholar	
Date of search	16/10/23	3/12/23		16/10/23		16/10/23		18/10/23	18/10/23	
Filters and limiters	Meta-Analysis Systematic review Child: Birth–18 years	MeSH descripted: [Rehabilitation] Child Health	MeSH descripted: [Rehabilitation]	Meta analysis OR Systematic review All child (0 to 18 years)	Meta analysis OR Systematic review	Meta analysis OR Systematic review	Meta analysis OR Systematic review	Meta analysis OR systematic review		
Search terms	Rehabilitation [MeSH Terms] Inpatient* OR tertiary OR hospital* OR ward*		paediatric* or pediatric* or infant* or child*	Rehabilitation OR rehab* inpatient* or tertiary or hospital* or ward*	Rehabilitation OR rehab* inpatient* or tertiary or hospital* or ward* p?ediatric* OR infant* OR child*	MH “Rehabilitation) OR rehab* paediatric* or pediatric* or infant* or child* inpatient* or tertiary or hospital* or ward*	MH “Rehabilitation, Pediatric”	Rehabilitation or rehab* p?ediatric* or infant* or child* inpatient* or tertiary or hospital* or ward*	(pediatric | paediatric) rehabilitation interventions “systematic review”	(“pediatric rehabilitation” | “paediatric rehabilitation”) interventions “systematic review”
Notes									First 50 results for each search included.	
Number of papers found	447	402		179		111		388	91	

### Eligibility of studies

We included systematic reviews (SRs), including meta-analyses, published from 1990 onwards. Eligible SRs had to:

- Include patients aged between 1 month and 18 years, with specific analysis available for these patients.
- Include papers explicitly set in the inpatient setting.
- Evaluate the efficacy of a medical or Allied Health intervention for neurological injury or insult.

SRs were excluded if they:

- Did not evaluate interventions occurring within 6 months of neurological injury or insult. Papers including only chronic conditions such as cerebral palsy or congenital conditions were deemed to fall into this category.
- Focused on cancer, cardiac, or pulmonary conditions, unless specifically evaluating an intervention for a neurological insult or injury outside of the expected evolution of the primary condition.
- Focused on complex pain, primary psychiatric, or functional disorders.
- Focused on rehabilitation following orthopaedic surgery, burns, or amputations.
- If the intervention studied was unclear.
- No full text available.
- Focused on interventions in acutely unstable patients in the period shortly after injury. For clarity, SRs with interventions occurring in intensive care settings were excluded, unless it was explicitly stated that patients were stable enough for management on less intensive wards. This exclusion criterion was added early in the review process in order to restrict paper selection to be within the scope of the review objective.

### Data collection

Titles and abstracts were screened by 2 reviewers, with a third reviewer available to resolve disputes. Papers of potential relevance were identified. This included some papers that had titles but no abstracts readily available. Full texts were obtained. The full texts were appraised by 2 reviewers, with a third reviewer available to resolve disputes.

The intention was that for each selected SR, overall objectives, paediatric population information, interventions, and outcomes for paediatric groups would be extracted using a predefined data extraction form.

### Assessment of quality and bias

SRs would be evaluated for quality using the AMSTAR 2 (A Measurement Tool to Assess Systematic Reviews) tool (12) and for bias with the ROBIS (Risk Of Bias In Systematic reviews) tool (13). Finally, selected SRs would be mapped onto the ICF framework (the International Classification of Functioning, Disability and Health) (14).

## RESULTS

**Fig. 1:**
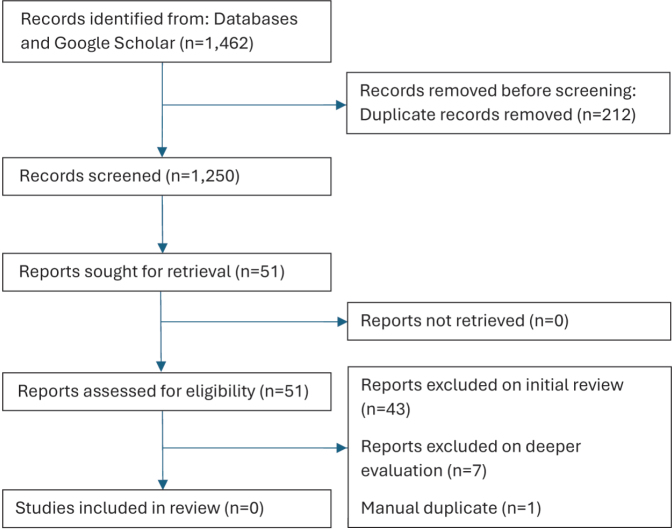
Selection process.

The electronic search identified 1,462 papers in total. These were uploaded into RefWorks (https://refworks.proquest.com/), after which 212 duplicates were omitted. A single further duplicate was manually identified. After the title and abstract screen, 51 papers underwent full text evaluation. Where an SR appeared promising but there was some ambiguity regarding whether it would meet the selection criteria, the papers reviewed by the SR were accessed for clarification. None of the selected papers met the selection criteria (see Fig. 1).

### Explanations of exclusions

Exclusions often were due to injury occurring more than 6 months before intervention or chronic conditions being investigated, an outpatient setting for intervention delivery, no paediatric patients included or no paediatric sub-analysis possible from the text. A summary of reasons for exclusion for papers that underwent full text review is in Table II. There were 8 SRs that were initially expected to meet selection criteria and were excluded on further evaluation.

**Table II:** Exclusions on full text review

Paper	Reference	Reason for exclusion
Cuello-Garcia et al., 2018*	(28)	Not inpatient rehabilitation OR No explicit subacute period intervention analysis
Freire et al., 2023	(39)	Not inpatient rehabilitation OR No explicit subacute period systematic intervention analysis
Getz et al., 2006	(40)	Chronic conditions/Over 6 months since injury
Deng et al., 2018	(41)	Chronic conditions/Over 6 months since injury
Cibinello et al., 2023	(42)	Chronic conditions/Over 6 months since injury
Mirkowski et al., 2019	(43)	Chronic conditions/Over 6 months since injury
Laufer et al., 2011	(44)	Chronic conditions/Over 6 months since injury
Ross et al., 2011#	(32)	Chronic conditions/Over 6 months since injury
Nguyen et al., 2021	(45)	Chronic conditions/Over 6 months since injury
Parsons et al., 2009	(46)	Chronic conditions/Over 6 months since injury OR intervention not targeted at neurorehabilitation
Morgan et al., 2012	(31)	Chronic conditions/Over 6 months since injury
Lukens et al., 2014	(47)	Chronic conditions/Over 6 months since injury
Rutkowski et al., 2020#	(25)	Chronic conditions/Over 6 months since injury
Getz et al., 2006*	(40)	Chronic conditions/Over 6 months since injury
Clapp et al., 2019	(48)	Chronic conditions/Over 6 months since injury
Shen et al., 2020#	(21)	Chronic conditions/Over 6 months since injury OR Outpatient setting OR Insufficient information
Galvin et al., 2011	(49)	Chronic conditions OR cannot differentiate between chronic and acquired cases
Jurdi et al., 2018#	(16)	Does not evaluate efficacy of interventions AND setting and chronicity unclear
Posadzki et al., 2013	(50)	Only chronic OR non-neurological conditions included
Treurnicht Naylor et al., 2011	(51)	Patients either chronic/injury >6 months ago OR efficacy of interventions not satisfactorily quantifiably evaluated
Morgan et al., 2008	(52)	Patients either outpatients OR Chronic conditions/Over 6 months since injury
Lindsay et al., 2015#	(15)	Patients either outpatients OR Chronic conditions/Over 6 months since injury
Jones et al., 2005	(53)	No Intervention evaluation
Hartman et al., 2015	(54)	No appraisal of efficacy of intervention
Kaelin et al., 2021	(55)	No appraisal of efficacy of intervention
Scurlock-Evans et al., 2014	(56)	No efficacy of intervention appraisal
Bagagiolo et al., 2016	(57)	No inpatient neurorehabilitation interventions in age group
Feng et al., 2021	(58)	No rehabilitation intervention appraised
Knight et al., 2019#	(4)	No systematic review directed at efficacy of appraised interventions
Mahan et al., 2017	(33)	Not inpatient setting
Franke et al., 2022	(59)	Not inpatient rehabilitation
Bogner et al., 2022*#	(17)	Not inpatient setting
Stocchetti et al., 2010	(60)	Not systematic review
Sturrock et al., 2017	(61)	Oral presentation
Lindsay et al., 2015	(62)	Oral presentation
Bragg et al., 2019	(63)	Outpatient setting AND scoping review
Fuke et al., 2018	(64)	No paediatric patients
Wang et al., 2019	(65)	No paediatric patients
Synnot et al., 2017	(66)	No paediatric patients
Cahill et al., 2020	(67)	No paediatric patients
Wong et al., 2013	(68)	No paediatric sub-analysis
Williamson et al., 2018	(69)	No paediatric sub-analysis
Harvey et al., 2017	(70)	No paediatric sub-analysis
Christie et al., 2018#	(18)	No paediatric sub-analysis OR Insufficient information
Lorusso et al., 2022	(71)	No paediatric patients
Song et al., 2022	(72)	No paediatric sub-analysis AND Retracted article
Law et al., 2018	(73)	No paediatric sub-analysis
Jiang et al., 2021	(74)	No paediatric patients
Glickman et al., 2010	(75)	Only chronic conditions for paediatric papers

* Abstract was not available at the time of title and abstract review.

# Reasons for exclusion are elaborated upon in the main body of the text.

“OR” indicates when different papers or patients within a review were excluded for different reasons.

Lindsay et al. (15) is an SR of interventions aimed at hospital-to-school transition. It was excluded as all reviewed papers either concerned interventions delivered as an outpatient or more than 6 months post-injury.

Jurdi et al. (16) is an SR on how game technologies are being used to improve children’s experience in hospital. It is largely descriptive regarding how game technologies are being used. It does not evaluate the efficacy of rehabilitation-relevant interventions in a way that is meaningful for this umbrella review. Information on chronicity and setting of interventions is also unclear.

Bogner et al. (17) is an SR looking at the effects on children with brain injuries’ behaviour of interventions targeted at parents. It evaluates interventions delivered online, as well as interventions traditionally associated with outpatient therapy that do not have the setting explicitly stated. Also, the time from injury when the interventions were delivered are unclear.

Christie et al. (18) is an SR looking at rehabilitation interventions for patients with encephalitis. It includes 3 papers that it reports as being focused on paediatrics. The only paediatric patient in Rao and Costa (19) was 19 years old, and as such does not meet this umbrella review’s eligibility criteria. The nature of the interventions investigated in both Tailor et al. (20) and Moorti et al. (76) are unclear both in Christie et al. and the papers themselves. No specific paediatric sub-analysis is available for Moorthi et al.

Knight et al. (4) is an SR of rehabilitation guidelines for children with both moderate and severe acquired brain injury. It reviewed whether guidelines include the presence and the quality of evidence available for recommendations. There is no systematic review of the evidence for the interventions themselves, so this paper did not meet our selection criteria. However, given some overlap in goals, further comment is made in our discussion.

Shen et al. (21) is an SR aiming to determine the effectiveness of virtual reality based paediatric traumatic brain injury rehabilitation. It included 3 papers of relevance. Tatla et al. (22) actually includes patients that meet our selection criteria, but did not meet Shen’s. This was because Shen focused on traumatic brain injury only. The patient that Shen did extract from Tatla did not meet our selection criteria as the intervention occurred over 6 months post-injury. Burdea et al. (23) reported on interventions performed in the outpatient setting. There was insufficient information available regarding Palma et al. (24) The original paper was unable to be sourced, and at time of writing the authors had not responded to a request via ResearchGate to supply it.

Rutkowski et al. (25) analysed the effectiveness of virtual reality in several fields of rehabilitation. Two paediatric patients were included, 1 with developmental coordination disorder and 1 with cerebral palsy, so were excluded due to these being chronic conditions.

A few further comments on exclusions follow.

Some other papers assessing transition to school were excluded when it was identified that all interventions took place (either explicitly or implicitly) after discharge (15, 26, 27).

Additionally, a decision was made to exclude papers that focussed on the intensive care setting where it was unclear whether the patients had been sufficiently medically stabilized to be usefully considered under a rehabilitation framework (28).

Meyling et al. (29) was identified outside of our search as being of relevance. It was not incorporated as it is a scoping review, which despite having parallel aims is not considered a systematic review (30).

## DISCUSSION

This umbrella review highlights a significant gap in systematically reviewed evidence supporting inpatient neurorehabilitation interventions for paediatric patients during the subacute period following neurological injury. Our comprehensive search strategy aimed to maximize sensitivity while relying on predefined selection criteria to ensure specificity. As anticipated, this approach yielded a large number of initial results. Despite this, none of the systematic reviews identified met our selection criteria. The findings emphasize the challenges in building a robust evidence base in this field, a trend also noted in other reviews of paediatric neurorehabilitation, where evidence supporting specific interventions is frequently lacking.

Several recurrent themes emerged from our exclusion criteria. The common reasons for exclusion were interventions not being performed in the inpatient setting, interventions targeting chronic rather than subacute conditions, and reviews focused on adult patients. This pattern reflects broader issues in the literature, where paediatric-specific interventions are often not adequately studied.

### Positioning our review within the wider literature

Our findings align with observations from other reviews in the field. A scoping review on subacute rehabilitation for acquired brain injury in children with some criteria overlapping with this review did not find any SRs to include. With regard to the inpatient setting, only cohort studies without concurrent controls were found (29). Other SRs struggled to find robust evidence for paediatric neurorehabilitation interventions. For example, a review on interventions for dysarthria following acquired brain injury (ABI) failed to identify studies focusing on the subacute inpatient period (31). Similarly, a review of psychosocial interventions post-ABI found no studies where interventions were delivered within 6 months of injury (32). Christie et al., discussed earlier, failed to find usable studies pertaining to paediatric encephalitis rehabilitation (18). Mahan et al., also discussed earlier, searched for prospective memory impairments and failed to find inpatient paediatric papers, as well as concluding that there is a lack of research overall relevant to their goals (33). These examples reinforce the broader trend of limited evidence available to guide clinical practice in paediatric neurorehabilitation.

The Knight et al. review of guidelines for inpatient paediatric rehabilitation further supports that weak evidence underpins current clinical recommendations. It found that many guidelines are based on expert opinion rather than empirical data, highlighting the need for more rigorous research to inform best practices (4). This issue is not confined to the inpatient setting. For example, a review of guidelines for managing mild acquired brain injury (ABI) in children similarly found that most recommendations lacked a solid evidence base, further reflecting the broader challenge of evidence generation in paediatric neurorehabilitation (34).

The reasons for the lack of SRs with positive findings cannot be conclusively determined from our results. A paucity of SRs is a contributor. However, given that reviews with overlap in scopes either failed to find relevant papers or found only low-quality evidence such as case series, the greater deficiency may be at the level of primary research. Breaking the field into domains and conducting scoping reviews may provide valuable additional insight to clarify where gaps lie.

### Further potential reasons for review outcome

Paediatric neurorehabilitation is a high-complexity, low-volume field. The relative rarity of paediatric neurological injuries requiring inpatient rehabilitation may reduce both research feasibility and funding interest. Although neurological injuries constituted 71% of inpatient paediatric rehabilitation admissions in Australasia, the absolute incidence requiring such care remains low at approximately 0.9 per 100,000 population (35–37). It was not possible to obtain complete international figures. It is also possible that numbers of centres providing inpatient paediatric rehabilitation are low, limiting opportunities for admission and research. Further work is needed to determine the burden of paediatric patients who might benefit from inpatient neurorehabilitation and the numbers and capacities of facilities available internationally to provide care.

### Limitations

A key limitation of this review was the exclusion of outpatient and online interventions, many of which could feasibly also be delivered in an inpatient setting. This focus was necessary to maintain the review’s scope, but future research should consider including studies where the setting is not a defining factor, to capture a broader range of relevant data. Likewise, reviewing the adult and chronic condition literature for translatable interventions was outside the scope of this review.

### Practical implications and opportunities for future research

In the first instance, the extent and research level of the deficiency should be better demarcated, such as through a series of scoping reviews.

Subsequently, ideally, generating more high-quality research in the inpatient setting would provide a stronger foundation for clinical practice. However, the diverse range of conditions and severities, the lack of universal outcome measures, and the absence of consensus on discharge endpoints complicate the feasibility of conducting randomized control trials and large-scale observational studies. A key priority for future research should be achieving greater uniformity and consensus on the use of existing outcome measures across different centres. This would facilitate more consistent evaluation of interventions and enhance the comparability of study results. This calls for international collaboration.

In the adult neurorehabilitation space, there is a growing shift towards community-based care models that emphasize rehabilitation outside of inpatient settings. A recent model of care for adult rehabilitation integrates therapy-led interventions with community resources to maximize patient recovery (38). While this shift offers potential benefits, it also highlights the need for a flexible model of care in paediatric neurorehabilitation, one that may need to rely more heavily on outpatient evidence where inpatient data are insufficient. Parallel movements in paediatrics are gaining traction, with an increasing interest in optimizing hospital to school and community transitions being 1 example (15, 26, 27).

Given the challenges in generating new high-quality evidence in paediatric inpatient neurorehabilitation, it is crucial, first, to carefully consider which potentially important interventions could feasibly be studied through RCTs and high-quality observational studies. In parallel, future efforts should focus on consolidating and leveraging existing data, including evidence from outpatient and hybrid rehabilitation models. A coordinated international effort, potentially involving a consortium dedicated to paediatric neurorehabilitation research, could help to overcome some of the barriers currently limiting progress in this field.

In summary, this review highlights possible gaps in the evidence surrounding paediatric inpatient neurorehabilitation in the subacute setting. While the challenges are considerable, a combination of targeted research, multidisciplinary approaches, and reliance on emerging evidence from outpatient settings may provide a way forward. Addressing these gaps will be essential for improving clinical outcomes and optimizing the care provided to paediatric patients during this critical phase of recovery.
